# Suicide in Chinese Graduate Students: A Review From 2000 to 2019

**DOI:** 10.3389/fpsyt.2020.579745

**Published:** 2020-12-04

**Authors:** Yun Cheng, Xiao Meng Zhang, Shui Ying Ye, Hui Min Jin, Xiu Hong Yang

**Affiliations:** Division of Nephrology, Pudong Medical Center, Shanghai Pudong Hospital, Fudan University, Shanghai, China

**Keywords:** suicide, China, graduate students, academic pressure, graduation pressure

## Abstract

Suicide is an important public problem in China. The characteristics of Chinese graduate students' suicides and the reasons they occur have never been reported systematically. We conducted a systematic search of public reports on local media and medical websites in this review to gain a basic understanding of these questions. A total of 150 cases of graduate students' suicides were reported from 2000 to 2019. Among the 150 students, 65.8% were male, nearly half between 26 and 30 years old, most (83.3%) never married, and 43.4% of graduation students committed suicide in graduation year and postponed years. The top three suicide methods were jumping, hanging, and drowning. Graduation pressure, depression, and academic pressure were the three leading suicidal causes. There is an urgent need for the Chinese government and universities to pay more attention to prevent suicides among graduate students.

## Introduction

Suicide is a major and serious public health problem worldwide. Nearly 800,000 people commit suicide each year, that is, one person every 40 s (1). Suicide occurs throughout the lifespan, is the second leading cause of death among people aged 15–34 years old globally, and accounts for 19% of all deaths (2).

Statistics showed that the Chinese suicide rate of people aged 25–39 years old in urban areas was about 2.35–2.87 per 100,000 people, as well as 4.47–4.91 in rural areas in 2018 (3). In recent years, the frequent suicides of Chinese graduate students have aroused heated social discussions. Graduate students have a high level of knowledge and a good psychological quality, but the suicides have made it necessary for us to re-understand this problem. However, there are no subgroup analyses of national suicide rates of different populations due to limits of the Chinese vital registration system, although the number of graduate students in this age group is facing a huge increase and the total enrollment reached 2.86 million in 2019 (4). Reports of graduate students' suicides hit headlines at times, and the concern for the mental health of these students has been enhanced. Although graduate students experience high levels of stress and anxiety and may be at an elevated risk for suicide, they receive less attention compared with college students, and there are few surveys of suicide in Chinese graduate students (5–7). A study involving 21,702 graduate students conducted in 2007 indicated that the prevalence of psychological distress for graduate students was about 6.8%, while the overall prevalence of suicide ideation among graduate students was about 1.78% (8).

The baseline characteristics for Chinese graduate students' suicide in the real world are still unknown. The purpose of this review is to summarize the cases of Chinese graduate students' suicides from 2000 to 2019, and we hope to provide a basic understanding of the characteristics of this subgroup of young people in China, summarize the death cases, and try to analyze them. Finally, the article aroused people's attention to the postgraduate group and tried to avoid the factors leading to suicide.

## Methods

We conducted a systematic search of public reports on local media, medical websites, Baidu (the most popular search engine in China), announcements on the official website of universities and scientific research institutions, and articles published in PubMed, CNKI (China National Knowledge Infrastructure), EBSCO (E.B. Stephens Company), Wanfang, and Weipu during 2000–2019 using the following search terms: “graduate students' suicide,” “Ph.D. suicide,” “Master's degree (MD) suicide,” “graduate students' jumping from building,” “Ph.D. jumping from building,” and “MD jumping from building.” Articles included were published in either English or Chinese. Baseline characteristics including sex, age, marital status, academic year, school level, school distribution, discipline category, and suicide methods and causes were extracted along with as much information as possible from reports.

## Results

We identified 150 suicide cases including 94 (62.6%) MD and 56 (37.3%) Ph.D. students from 2000 to 2019. Baseline characteristics (sex, age, marital status, academic year, school level, school distribution, and discipline category) of graduate student's suicides, as well as suicide methods and causes of suicides are summarized in Table 1.

**Table 1:** Baseline characteristics of MD and Ph.D. students' suicides.

	**Total (*N* = 150)**	**Master's degree (*N* = 94)**	**Doctor's degree (*N* = 56)**
**Sex**, ***n*** **(%)**			
Male	98 (65.3%)	54 (57.4%)	44 (78.6%)
Female	52 (34.7%)	40 (42.6%)	12 (21.4%)
**Age (years old)**, ***n*** **(%)**			
20–25	47 (31.3%)	28 (29.8%)	19 (33.9%)
26–30	74 (49.3%)	57 (60.6%)	17 (30.4%)
31–35	10 (6.7%)	2 (2.1%)	8 (14.3%)
36–40	5 (3.3%)	1 (1.1%)	4 (7.1%)
**Marital status**, ***n*** **(%)**			
Married	13 (8.7%)	3 (3.2%)	10 (17.9%)
Never married	125 (83.3%)	89 (94.7%)	36 (64.3%)
Divorced	1 (0.7%)	0 (0.0%)	1 (1.8%)
**Academic year**, ***n*** **(%)**			
1st	29 (19.3%)	24 (25.5%)	5 (8.9%)
2nd	42 (28.0%)	31 (33.0%)	11 (19.6%)
3rd	52 (34.7%)	33 (35.1%)	19 (33.9%)
Postponed^a^	13 (8.7%)	2 (2.1%)	11 (19.6%)
**School level**			
Key university^b^	115 (76.7%)	70 (74.5%)	45 (80.4%)
Non-key university	20 (13.3%)	20 (21.3%)	0 (0%)
Foreign universities	15 (10.0%)	4 (4.2%)	11 (19.6%)
**School distribution^c^**			
Eastern region	92 (61.3%)	58 (61.7%)	34 (60.7%)
Northeast region	8 (5.3%)	6 (6.4%)	2 (3.8%)
Central region	22 (14.7%)	14 (14.9%)	8 (14.3%)
Western region	10 (6.7%)	9 (9.6%)	1 (1.8%)
Foreign	15 (10.0%)	4 (4.2%)	11 (19.6%)
**Discipline category**			
Engineering	49 (32.7%)	7 (7.4%)	42 (75.0%)
Science	37 (24.7%)	36 (38.3%)	1 (1.8%)
Literature	13 (8.7%)	13 (13.8%)	0 (0%)
Medicine	11 (7.3%)	11 (11.7%)	0 (0%)
Other^d^	24 (16.0%)	11 (11.7%)	13 (23.2%)
**Suicide method**			
Jumping	98 (65.3%)	61 (64.9%)	37 (66.1%)
Hanging	26 (17.3%)	14 (14.9%)	12 (21.4%)
Drowning	11 (7.3%)	6 (6.4%)	5 (8.9%)
Other^e^	15 (10.0%)	13 (13.8%)	2 (3.6%)
**Cause of suicide**			
Graduation pressure	31 (20.7%)	18 (19.1%)	13 (23.2%)
Depression	27 (18.0%)	20 (21.3%)	7 (12.5%)
Academic pressure	13 (8.7%)	8 (8.5%)	5 (8.9%)
Failed love affair	12 (8.0%)	9 (9.6%)	3 (5.4%)
Other^f^	21 (14.0%)	12 (12.8%)	9 (16.1%)

a *Graduates can extend their graduation for up to 8 years*.

b *Universities from 112 national key universities that belong to the “211 project” and research institutions*.

c *Based on China's economic and social situation, the country is divided into four major economic regions: the eastern, northeast, central, and western regions*.

d *Including agriculture, economics, education, history, art, law, management, each of which had no more than five suicided students*.

e *Including gas suicide, burning oneself, cutting, taking poison, charcoal-burning, electrocution, each of which accounted for no more than four suicided students*.

f *Including family conflict, employment pressure, financial stress, interpersonal conflict, disease, each of which accounted for no more than four suicided students*.

Among the 150 students, male students accounted for 57.4% (*n* = 54) and 78.4% (*n* = 44) in MD and Ph.D. students, respectively. Almost half of graduate students (49.3%, *n* = 74) were between 26 and 30 years old. Most graduate students (83.3%, *n* = 125) were never married, 8.7% (*n* = 13) were married, and only 0.7% (*n* = 1) were divorced (Table 1).

About a third of graduate students (34.7%, *n* = 52) tended to commit suicide in graduation year, followed by second year (28.0%, *n* = 42), first year (19.3%, *n* = 29), and postponed year (8.75%, *n* = 13). About three-quarters (76.7%, *n* = 115) were from key universities, followed by non-key universities (13.3%, *n* = 20) and foreign universities (10.0%, *n* = 15) (Table 1).

There were three main suicide methods: jumping (65.3%, *n* = 98), hanging (17.3%, *n* = 26), and drowning (7.3%, *n* = 11). The first three suicide methods among MD were jumping (64.9%, *n* = 61), hanging (14.9%, *n* = 14), and drowning (6.4%, *n* = 6). As to the Ph.D. students, the main suicide methods were similar to MD students. Graduation pressure (20.7%, *n* = 31), depression (18.0%, *n* = 27) and academic pressure (8.7%, *n* = 13) were the leading causes of suicide among all 150 students. The top three reasons that MD committed suicide were depression (21.3%, *n* = 20), graduation pressure (19.1%, *n* = 18), and love affairs (9.6%, *n* = 9) (Table 1).

Baseline characteristics of male and female students' suicides are also summarized in Table 2. More male students than female students committed suicide among 26–30 year old students (56.1 vs. 36.5%), which was consistent with the findings: more male students got married (11.2 vs. 3.8%) and entered the Ph.D. program (44.9 vs. 23.1%). Almost half of the male students (48.9%) committed suicide in graduation year and postponed year, and this was in accordance with the most common suicide reason of male students: graduation pressure (26.5%), while our research showed female students committed suicide for depression (28.8%). Besides, male students tended to study in better universities and research institutes like key universities and foreign universities (89.8%) and choose more science and engineering than female students (62.2 vs. 48.1%) (Table 2).

**Table 2:** Baseline characteristics of male and female students' suicides.

	**Total (*N* = 150)**	**Male (*N* = 98)**	**Female (*N* = 52)**
**Age (years old)**, ***n*** **(%)**			
21–25	47 (31.3%)	24 (24.5%)	13 (25.0%)
26–30	74 (49.3%)	55 (56.1%)	19 (36.5%)
31–35	10 (6.7%)	6 (6.1%)	4 (7.7%)
36–40	5 (3.3%)	3 (3.1%)	2 (3.8%)
**Marital status**, ***n*** **(%)**			
Married	13 (8.7%)	11 (11.2%)	2 (3.8%)
Never married	125 (83.3%)	79 (80.6%)	46 (88.5%)
Divorced	1 (0.7%)	0 (0.0%)	1 (1.9%)
**Students' degree**, ***n*** **(%)**			
Master's degree	94(62.7%)	54 (55.1%)	40 (76.9%)
Doctor's degree	56(37.3%)	44 (44.9%)	12 (23.1%)
**Academic year**, ***n*** **(%)**			
1st	29 (19.3%)	16 (16.3%)	13 (25.0%)
2nd	42 (28.0%)	27 (27.6%)	15 (28.8%)
3rd	52 (34.7%)	36 (36.7%)	16 (30.8%)
Postponed^a^	13 (8.7%)	12 (12.2%)	1 (1.9%)
**School level**			
Key university^b^	115 (76.7%)	77 (78.6%)	38 (73.1%)
Non-key university	20 (13.3%)	10 (10.2%)	10 (19.2%)
Foreign universities	15 (10.0%)	11 (11.2%)	4 (7.7%)
**School distribution^c^**			
Eastern region	92 (61.3%)	61 (62.2%)	31 (59.6%)
Northeast region	8 (5.3%)	5 (5.1%)	3 (5.8%)
Central region	22 (14.7%)	14 (14.3%)	8 (15.4%)
Western region	10 (6.7%)	7 (7.1%)	3 (5.8%)
Foreign	15 (10.0%)	11 (11.2%)	4 (7.7%)
**Discipline category**			
Engineering	49 (32.7%)	35 (35.7%)	14 (26.9%)
Science	37 (24.7%)	26 (26.5%)	11 (21.2%)
Literature	13 (8.7%)	6 (6.1%)	7 (13.5%)
Medicine	11 (7.3%)	6 (6.1%)	5 (9.6%)
Other^d^	24 (16.0%)	16 (16.3%)	8 (15.4%)
**Suicide method**			
Jumping	98 (65.3%)	68 (69.4%)	30 (57.7%)
Hanging	26 (17.3%)	17 (17.3%)	9 (17.3%)
Drowning	11 (7.3%)	6 (6.1%)	5 (9.6%)
Other^e^	15 (10.0%)	7 (7.1%)	8 (15.4%)
**Cause of suicide**			
Graduation pressure	31 (20.7%)	26 (26.5%)	5 (9.6%)
Depression	27 (18.0%)	12 (12.2%)	15 (28.8%)
Academic pressure	13 (8.7%)	7 (7.1%)	6 (11.5%)
Failed love affair	12 (8.0%)	8 (8.2%)	4 (7.7%)
Other^f^	21 (14.0%)	15 (15.3%)	6 (11.5%)

a *Graduates can extend their graduation for up to 8 years*.

b *Universities from 112 national key universities that belong to the “211 project” and research institutions*.

c *Based on China's economic and social situation, the country is divided into four major economic regions: the eastern, northeast, central, and western regions*.

d *Including agriculture, economics, education, history, art, law, management, each of which had no more than five suicided students*.

e *Including gas suicide, burning oneself, cutting, taking poison, charcoal-burning, electrocution, each of which accounted for no more than four suicided students*.

f *Including family conflict, employment pressure, financial stress, interpersonal conflict, disease, each of which accounted for no more than four suicided students*.

## Discussion

According to statistics found in this survey during the past two decades from 2000 to 2019, 94 MD and 56 Ph.D. students committed suicide. Why are these students prone to committing suicide? We list several reasons for consideration.

### Male Students Bear More Economic and Social Pressure

In this review, we found that there were more male than female graduate students, both master's and Ph.D. students, who committed suicide. Males have a higher risk of completed suicide than females in western countries. While in China, females' suicide rate used to be much higher than males; according to a meta-analysis of 192,362 subjects, females had a 2.35-fold higher risk of completed suicide than males (9). Things are very different now; recent statistics show that the female suicide rate decreased much more than the male suicide rate from 1987 to 2008 (20.4–6.2/100,000 vs. 14.9–7.0/100,000, respectively), and the suicide rate of young males exceeded young females around 2010 because of economic growth and urbanization (10).

Chinese men are often considered to be the main breadwinners in the family, and both the family and society place high demands on them. The preponderance of males in completed suicides is known as the “gender paradox of suicidal behavior,” which is very obvious in these male students (11). As Table 2 shows, male graduation students were older, went into higher and better education institutions, got married more, and bore more graduation pressure; all these characteristics might have brought them much pressure from both their families and society. But Chinese young men are required to be tolerant in traditional culture, and mental problems are considered shameful, so it is not easy to seek help from one's social network or psychiatrists for male graduation students.

### Graduating on Time Becomes Harder

The relationship between education level and suicide has long been discussed. One report analyzed data from nationally representative data on the prevalence and risk factors of attempted suicide in the National Comorbidity Survey, indicating that being poorly educated is a significant risk factor that was strongly related to suicide ideation (12). Some American psychiatrists who examined the risk factors associated with each stage of life suggested that low education level was an important risk factor of adult suicide (13). One study assessed the complex impact of risk and protective factors on suicide mortality in the Ukrainian general population and suggested that education was negatively associated with suicide (14). However, the link between education levels and suicide rates still remains controversial.

Nature's survey of more than 5,700 doctoral students worldwide conducted in 2017 revealed that, although Ph.D. students love what they do, many also suffer for it. Maintaining work-life balance, a career path, and financial issues are the top three concerns after starting their Ph.D. career, and roughly half of doctoral students respond with these worries (15). What about Chinese graduate students? We found that the leading cause of suicide in this population was graduation pressure, and doctoral students suffered more from graduation pressure. This is consistent with the finding that the largest proportion of these suicide cases happened in senior and postponed students, which also indicates that graduation pressure is the top pressure in these students.

Data from the Chinese Ministry of Education shows that the delayed graduation rate of academic doctorates remained above 60% from 2010 to 2018 (16). Delayed graduation undoubtedly causes psychological, social, and economic pressure on students. This may explain why graduate stress is the most common cause of suicide among graduate students.

### Depression Is Not Taken Seriously

According to Nature's survey of Ph.D. students, 690 responses from Chinese students showed that students in China faced strains that could threaten mental health. In this survey, 40% of Chinese respondents said that they had sought help for depression or anxiety caused by their Ph.D. program which was slightly more than the 36% of respondents among their foreign counterparts (17).

In our study, 18% (27 cases) of suicidal graduate students had a history of depression. The proportion of depression may be greatly underestimated because of inadequate reporting and Chinese people's stigma concerning mental illness. One study carried out in several central universities with a total of 5,972 undergraduate students showed that psychological distress and suicidal behavior were both common among university students, and psychological distress was highly associated with suicidal behavior (18). One meta-analysis provided more specific points: there was a moderate association between depressive symptoms and suicidal ideation among university students in China, and depressive symptoms contributed to the development of suicidal ideation (19). However, to our knowledge, there are no large-scale studies on the association between depression and suicide in Chinese graduate students.

### Physical and Mental Fatigue Caused by Failed Love Affair

Graduate students in China often have an intimate partner. In the highly stressful life of graduate students, communicating with close partners is often a good way to relieve stress. At the same time, maintaining this intimate relationship also consumes a certain amount of time and money for them. If a graduate student cannot feel the love from an intimate partner, or even the relationship becomes a burden, the graduate student is often very helpless and feels doubled pressure. When the suicide has the main reasons mentioned above, the failed love affairs often trigger suicidal thoughts.

There are several limitations in our review. First, our records of the MD and Ph.D. students' suicides may be incomplete and may underestimate the actual incidence. This is probably because some cases have not been reported in Chinese websites or local media. Second, we do not know these victims' medical histories, especially their mental health problems, because of the insufficiently detailed reports. Third, even in the cases reported publicly, some important information was not included, such as location of residence and family structure. Rural or urban, single-child family and single-parent family are always taken as significant factors that affect peoples' suicidal acts (9, 20). Last but not least, because part of the data of total male/female ratio and MD/Ph.D. ratio for the last 20 years were not available from government websites, *P* value cannot be compared statistically.

Overall, graduation pressure, depression, and academic pressure may be the top three causes that lead to Chinese graduate students' suicides. The purpose of this review was to summarize the cases of Chinese graduate students' suicide in the past two decades and summarize the basic characteristics. Through describing and analyzing the characteristics of graduate students' suicides, we hope to emphasize the seriousness of graduate students committing suicide and call for action to prevent more suicides among Chinese graduate students.

## Data Availability Statement

The original contributions presented in the study are included in the article/supplementary materials, further inquiries can be directed to the corresponding author/s.

## Author Contributions

XY and HJ conceived of and designed the research. XZ and SY collected and analyzed data. YC wrote the manuscript. All authors contributed to the article and approved the submitted version.

## Conflict of Interest

The authors declare that the research was conducted in the absence of any commercial or financial relationships that could be construed as a potential conflict of interest.
